# Coronatine Inhibits Stomatal Closure through Guard Cell-Specific Inhibition of NADPH Oxidase-Dependent ROS Production

**DOI:** 10.3389/fpls.2016.01851

**Published:** 2016-12-14

**Authors:** Laila Toum, Pablo S. Torres, Susana M. Gallego, María P. Benavídes, Adrián A. Vojnov, Gustavo E. Gudesblat

**Affiliations:** ^1^Instituto de Ciencia y Tecnología Dr. César Milstein, Fundación Pablo Cassará, Consejo Nacional de Investigaciones Científicas y TécnicasBuenos Aires, Argentina; ^2^Departamento de Química Biológica, Facultad de Farmacia y Bioquímica, Universidad de Buenos AiresBuenos Aires, Argentina; ^3^Instituto de Biodiversidad y Biología Experimental y Aplicada, Departamento de Biodiversidad y Biología Experimental, Consejo Nacional de Investigaciones Científicas y Técnicas, Facultad de Ciencias Exactas y Naturales, Universidad de Buenos AiresBuenos Aires, Argentina

**Keywords:** *Arabidopsis thaliana*, coronatine, NADPH oxidase, reactive oxygen species, stomata, abscisic acid

## Abstract

Microbes trigger stomatal closure through microbe-associated molecular patterns (MAMPs). The bacterial pathogen *Pseudomonas syringae* pv. *tomato* (*Pst*) synthesizes the polyketide toxin coronatine, which inhibits stomatal closure by MAMPs and by the hormone abscisic acid (ABA). The mechanism by which coronatine, a jasmonic acid-isoleucine analog, achieves this effect is not completely clear. Reactive oxygen species (ROS) are essential second messengers in stomatal immunity, therefore we investigated the possible effect of coronatine on their production. We found that coronatine inhibits NADPH oxidase-dependent ROS production induced by ABA, and by the flagellin-derived peptide flg22. This toxin also inhibited NADPH oxidase-dependent stomatal closure induced by darkness, however, it failed to prevent stomatal closure by exogenously applied H_2_O_2_ or by salicylic acid, which induces ROS production through peroxidases. Contrary to what was observed on stomata, coronatine did not affect the oxidative burst induced by flg22 in leaf disks. Additionally, we observed that in NADPH oxidase mutants *atrbohd* and *atrbohd/f*, as well as in guard cell ABA responsive but flg22 insensitive mutants *mpk3, mpk6, npr1-3*, and *lecrk-VI.2-1*, the inhibition of ABA stomatal responses by both coronatine and the NADPH oxidase inhibitor diphenylene iodonium was markedly reduced. Interestingly, coronatine still impaired ABA-induced ROS synthesis in *mpk3, mpk6, npr1-3*, and *lecrk-VI.2-1*, suggesting a possible feedback regulation of ROS on other guard cell ABA signaling elements in these mutants. Altogether our results show that inhibition of NADPH oxidase-dependent ROS synthesis in guard cells plays an important role during endophytic colonization by *Pst* through stomata.

## Introduction

Stomatal pores allow plants to exchange gases with the atmosphere but can also be used by phytopathogens to colonize leaves. As a result, plants have evolved the capacity to close stomata not only in response to hormones such as abscisic acid (ABA) and abiotic stresses, but also to microbe-associated molecular patterns (MAMPs), including bacterial flagellin, elongation factor Tu, lipopolysaccharide, fungal chitin, and yeast elicitor. Microbes in turn have evolved strategies to avoid closing stomata so as to colonize hosts via these openings (Arnaud and Hwang, 2014; McLachlan et al., 2014).

Stomatal closure in response to different stimuli is brought about by loss of turgor of guard cells, which is caused by the extrusion of solutes through different ion channels. These channels are regulated by a complex signaling network involving production of reactive oxygen species (ROS), nitric oxide, phospholipids, cytosolic calcium elevations, cytosolic alkalinization, and other signaling components (Kim et al., 2010; Joshi-Saha et al., 2011; Kollist et al., 2014). A minimal signaling cascade has been established for ABA-induced stomatal closure in the model plant *Arabidopsis thaliana*. ABA is perceived by receptors PYR/PYL/RCAR, which inhibit PP2Cs phosphatases, ABI1 and ABI2. In the absence of ABA these phosphatases act as inhibitors of the protein kinase OST1/SnRK2.6. Therefore ABA perception causes activation of this kinase, which phosphorylates and activates the anion channel SLAC1, an event that contributes to membrane depolarization and subsequent solute exit from guard cells (Cutler et al., 2010; Joshi-Saha et al., 2011). However, several other signaling components like those mentioned above have also been shown to be involved ABA-induced stomatal closure, indicating that signaling of this hormone likely occurs through a more complex signaling network.

The flagellin derived peptide flg22 is perceived by the RLK receptor complex FLS2-BAK1 and phosphorylates the kinase BIK1, which in turn activates by phosphorylation the NADPH oxidase AtRBOHD (Kadota et al., 2014; Li et al., 2014). This leads to ROS production, which in guard cells leads to activation of plasma membrane Ca^2+^ channels (Kwak et al., 2003; Thor and Peiter, 2014) and subsequently of SLAC1 anionic channel (Guzel Deger et al., 2015), causing stomatal closure. While *atrbohd* mutant is completely insensitive to flg22 or bacteria for stomatal closure, in the case of ABA, a double mutation in *AtRBOHD* and *AtRBOHF* leads only to a partial inhibition of stomatal closure triggered by this hormone, something likely due to the redundancy of the complex ABA signaling network (Kwak et al., 2003). NADPH oxidases are also involved in stomatal closure triggered by darkness (Desikan et al., 2004). Like *AtRBOHD*, mitogen activated kinases MPK3 and MPK6, which are activated by flg22 in mesophyll protoplasts, are required for stomatal closure triggered by bacterial MAMPs but not by ABA (Gudesblat et al., 2009; Montillet et al., 2013). Similarly, the MAMP-activated L-type lectin receptor kinase-VI.2 (LECRK-VI.2) is also required for stomatal closure triggered by bacterial MAMPs but not by ABA (Singh et al., 2013). Stomatal closure by certain MAMPs such as chitin and yeast elicitor, and also by the hormone salicylic acid (SA), involved in response against pathogens, requires ROS production by cell wall peroxidases. Therefore the action of these compounds is not affected in NADPH oxidase mutants, but it is by the peroxidase inhibitor salicylhydroxamic acid (SHAM; Khokon et al., 2010a,b, 2011). Functional SA signaling, involving the transcription factor NPR1, is required for stomatal closure induced by flg22 or bacteria but not by ABA (Zeng and He, 2010).

Several pathogens that penetrate leaves through stomata have evolved the capacity to produce compounds that prevent their closure. The bacterial pathogen *Pst* DC 3000 synthesizes coronatine, a polyketide toxin that mimics the active form of jasmonic acid (JA), a hormone involved in defense, jasmonoyl-L-isoleucine (JA-Ile; Yan et al., 2009; Geng et al., 2014), and which can reopen stomata after initial closure triggered by bacterial MAMPs, ABA, or darkness (Mino et al., 1987; Melotto et al., 2006; Egoshi et al., 2016; Panchal et al., 2016). The inhibitory effect of coronatine on stomata depends in *A. thaliana* on the presence of the JA receptor COI1 and the JA responsive transcription factors MYC2, ANAC019, ANAC055, and ANAC072 (Melotto et al., 2006; Zheng et al., 2012) and in tomato on the JA responsive transcription factor JA2L (Du et al., 2014). Thus, in order to inhibit stomatal closure coronatine requires the same signaling pathway as JA.

In order to gain knowledge into the mechanism of action of coronatine on stomata, in this work we investigated its effect on ROS production because these molecules act as important signaling hubs in guard cells. We found that this toxin inhibits ROS production mediated by NADPH oxidases but not by peroxidases, and does it specifically in stomata. Additionally, we observed that in *atrbohd*, *atrbohd/f*, *mpk3, mpk6, npr1-3*, and *lecrk-VI.2-1* mutants, all affected in response to MAMPs but not to ABA, coronatine is unable to inhibit the effect of ABA on stomata, even when it can still inhibit ABA-induced ROS synthesis.

## Materials and Methods

### Plant Material and Growth Conditions

Plants were grown in petri dishes containing half-strength Murashige and Skoog (MS) medium with 1% sucrose under a 12 h:12 h light/dark cycle (photon flux density of 90 μE) at 22–23°C. After a week plants were transferred to a mixture of vermiculite, peat moss and perlite (1:1:1). *A. thaliana* L. Heynh. ecotypes Columbia-0 (Col-0) or Landsberg *erecta* (Ler) were used as controls. The following *A. thaliana* mutants were used: *atrbohd*, *atrbohd/atrbohf (atrbohd/f*; Cao et al., 1997; Torres et al., 2002), *npr1-*3 (Cao et al., 1997), *mpk3* (SALK_151594), *mpk6* (SALK_127507; Alonso et al., 2003), *coi 1-16* (Ellis and Turner, 2002), *ost1-2* (Mustilli et al., 2002), *bik1* (Veronese et al., 2006), *lecrk-VI.2-1* (SALK_070801; Singh et al., 2012), and *pp2ca-1* (Kuhn et al., 2006).

### Chemicals

Abscisic acid (mixed isomers), 2′,7′-dichlorodihydrofluorescein diacetate (H_2_DCFDA), SHAM, SA, diphenylene iodonium (DPI), horseradish peroxidase, luminol, and coronatine were purchased from Sigma (USA), H_2_O_2_ from JT Baker (USA), while flg22 was synthesized by GL Biochem (China). ABA and SHAM were dissolved in ethanol, H_2_DCFDA in dimethyl sulfoxide and coronatine in methanol, keeping in all cases the final solvent concentration in assays below 1%.

### Stomatal Aperture Bioassays

Stomatal bioassays were performed as previously described (Gudesblat et al., 2009). Epidermal peels from leaves of 4-week-old plants were floated in 10:10 buffer under light (10 mM KCl and 10 mM MES-KOH, pH 6.15) for 2.5 h, then ABA (at the indicated concentrations), SA (10 μM), flg22 (5 μM), DPI (20 μM), SHAM (2 mM), or *Pst* DC3118 (10^8^ cfu/mL) were added to the medium and peels were incubated for a further 1.5 h. Coronatine (1.56 μM) was added 10 min previous to other treatments. For darkness-induced stomatal experiments, after initial opening epidermal peels were incubated in buffer 10/10 for 2 h in the dark in the presence or absence of chemicals. Promotion of closure by H_2_O_2_ was performed as previously described (Pei et al., 2000). The initial incubation to open stomata was performed in the presence of 0.1 mM EGTA, and then H_2_O_2_ was added (100 μM) together with CaCl_2_ (0.2 mM). Coronatine was added 10 min prior to the addition of other chemicals. For the inhibition of stomatal opening experiment, epidermal peels were floated in the dark in 10:0 buffer (10 mM MES-KOH pH 6.15) for 2 h to promote closure. Then epidermal peels were transferred to 10:10 buffer containing chemicals as described above, and were incubated in the light for an additional 2 h period. The aperture of 40 stomata was measured for each treatment. Data are presented as the average from 80 to 120 aperture measurements, collected from two to three independent experiments. Mock treatments were performed with 10:10 buffer.

### ROS Measurements

Hydrogen peroxide production in guard cells was measured using H_2_DCFDA (Murata et al., 2001). After a 2.5 h incubation in 10:10 buffer under light conditions, epidermal peels were transferred to a 10 mM Tris-HCl pH 7.2 buffer containing H_2_DCFDA (10 μM) for 15 min. Excess H_2_DCFDA was removed by washing three times with 10 mM Tris-HCl pH 7.2. Then peels were transferred to 10:10 buffer containing 5 μM flg22 or 20 μM ABA and incubated for 20 min in the dark. Coronatine (1.56 μM) was added 10 min previous to flg22 and ABA. Fluorescence was observed with a Nikon Eclipse E600 fluorescence microscope (excitation 460–480 nm, emission 495–540 nm). The guard cells fluorescence was analyzed using ImageJ 1.46 software. Data is presented as the average from 80 fluorescence measurements per treatment, collected from two independent experiments. Oxidative burst measurement in leaf disks was performed using the peroxidase luminol enhanced chemiluminescence method (Gimenez-Ibanez et al., 2009). ROS production was induced in 12 leaf disks per treatment from 4- to 5-week-old plants. Disks (0.25 cm^2^) were incubated overnight in water, which was replaced by 100 μl of 10 mM Tris/HCl pH 9.5 containing 10 μg/ml horseradish peroxidase and 20 μM luminol. ROS were elicited with flg22 (1 μM), and coronatine (1.56 μM) was added simultaneously with flg22 where indicated. The measurement of chemiluminescence was performed in a plate reader PHERAstar Plus HTS, BGM Labtech. Results are presented as the average from 36 fluorescence measurements per treatment, collected from three independent experiments. The superoxide anion scavenging activity of coronatine was determined by inhibition of adrenochrome formation rate at 480 nm as previously described (Misra and Fridovich, 1972). The reaction mixture contained 1 mM epinephrine (20 mM in 0.1 M HCl), 50 mM sodium carbonate buffer (pH 10.2) and different concentrations of coronatine. The increase in absorbance due to the formation of the adrenochrome was followed for 10 min and the activity was calculated from the linear part of the curve in absence or presence of coronatine. Ascorbic acid was used as positive control of superoxide scavenging activity. Absorbance was determined in a FlexStation 3 Multi-Mode Microplate Reader (Molecular Devices). Mock treatments were performed with the respective incubation buffer.

### Statistical Analysis

For statistical analysis, one-way ANOVA (*post hoc* Tukey’s test) or two-way ANOVA (Bonferroni’s method) tests were used. Statistical significances were determined with InfoStat software (InfoStat 2013 version).

## Results

### Effect of Coronatine on ROS Production in Guard Cells

**FIGURE 1:**
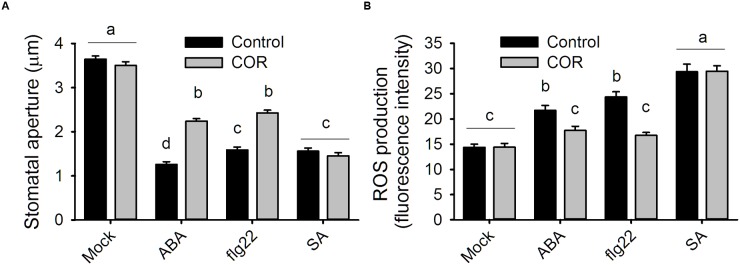
**Coronatine affects stomatal closure and ROS production by ABA and flg22 but not by SA.** **(A)** Coronatine (COR, 1.56 μM) inhibited stomatal closure in response to ABA (20 μM) or flg22 (5 μM) but not to SA (10 μM). Stomatal apertures were measured 1.5 h after application of the respective treatments. **(B)** Coronatine (COR, 1.56 μM) inhibited guard cell ROS production in response to ABA (20 μM) or flg22 (5 μM) but not to SA (10 μM). ROS were detected as H_2_DCFDA fluorescence in guard cells 20 min after treatment. In both experiments coronatine was added 10 min before other treatments. Different letters indicate significant differences at *p* < 0.05 in **(A,B**; one-way ANOVA, Tukey’s test). Error bars represent SE from two **(B)** to three **(A)** independent trials, *n* = 40 per trial in all experiments.

**FIGURE 2:**
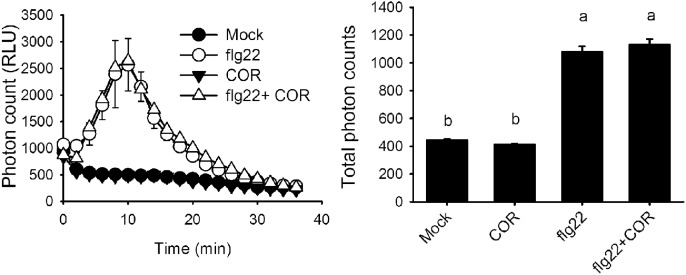
**Coronatine does not affect ROS induction by flg22 in leaf disks.** Coronatine (COR, 1.56 μM) did not prevent ROS induction by flg22 (1 μM) in leaf disks. ROS were measured by the peroxidase luminol enhanced chemiluminescence method and are expressed as relative light units (RLU). Values are averages of total photon counts from each treatment. Different letters indicate significant differences at *p* < 0.01 (one-way ANOVA, Tukey’s test). Error bars represent SE from three independent trials, *n* = 12 per trial.

It was previously reported that coronatine can reduce stomatal closure triggered by ABA and flg22 (Melotto et al., 2006). Measurement of ROS in *A. thaliana* guard cells revealed that coronatine strongly inhibited their synthesis in response to both compounds, which is mediated by NADPH oxidases. By contrast, the toxin failed to inhibit stomatal closure or ROS production induced by SA, which induces the synthesis of these second messengers through peroxidases (**Figures 1A,B**). ROS induction by SA was higher than that elicited by flg22 or ABA, however, that did not translate into stronger stomatal closure, suggesting that ROS signaling is saturated beyond a certain concentration of ROS. These results indicate that coronatine interferes with stomatal closure through inhibition of ROS production by NADPH oxidases but not by peroxidases. Consistent with previous reports showing that ROS induction by SA is mediated by peroxidases, the peroxidase inhibitor SHAM inhibited closure by SA but not by flg22, while SA was capable of closing stomata in the *atrbohd* mutant (**Supplementary Figure S1**). As flg22 induces NADPH-dependent ROS production in leaf disks, we tested whether coronatine can affect it and, we found that this is not the case (**Figure 2**), showing that the effect of this toxin on ROS production in stomata does not occur in other leaf tissues.

### The Inhibitory Effect of Coronatine on Stomata Requires NADPH Oxidases

**FIGURE 3:**
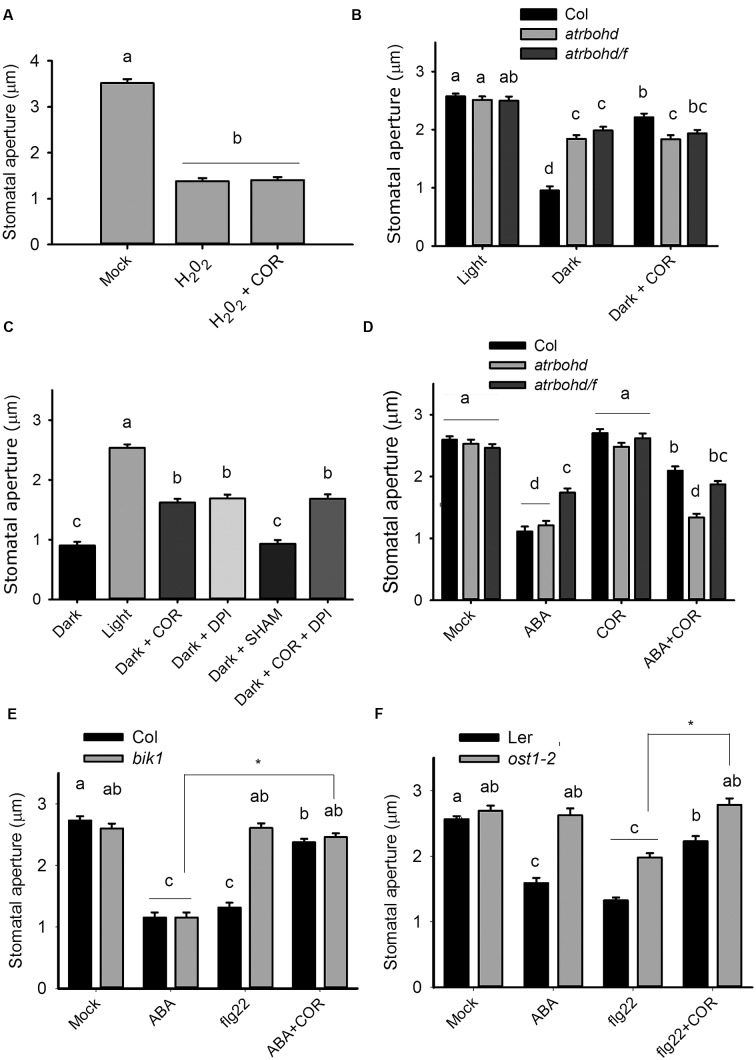
**The effect of coronatine on stomatal closure requires ROS production by NADPH oxidases but does not depend on OST1 and BIK1 kinases.** **(A)** Coronatine (COR, 1.56 μM) could not inhibit stomatal closure induced by H_2_O_2_ (100 μM). Stomatal apertures were measured 1.5 h after application of the respective treatment. **(B)** *atrbohd* and *atrbohd/f* mutants are partially impaired in darkness-induced stomatal closure and coronatine (COR, 1.56 μM) failed to further inhibit it. **(C)** Coronatine (COR, 1.56 μM), DPI (20 μM) but not SHAM (2 mM) inhibited darkness-induced stomatal closure **(D)** Coronatine (COR, 1.56 μM) failed to inhibit promotion of closure by ABA (20 μM) in *atrbohd* and *atrbohd*/*f* mutants but not in Col. **(E)** *bik1* mutant is sensitive to coronatine (COR, 1.56 μM) for ABA-induced stomatal closure. **(F)** *ost1-2* mutant is sensitive to coronatine (COR, 1.56 μM) for flg22-induced stomatal closure. In **(B**,**C)** coronatine, DPI or SHAM were added at the beginning of the incubation with buffer 10/10 for 2 h. In **(A)** and **(D–F)** coronatine was added 10 min before the respective treatments. Different letters indicate significant differences at *p* < 0.05 in **(B)** and **(D–F**; two-way ANOVA) and **(A,C**; one-way ANOVA, Tukey’s test). Datasets marked with asterisks are significantly different as assessed by ANOVA test, ^∗^*p* < 0.05. Error bars represent SE from two **(A,B)** and **(D–F)** or three **(C)** independent trials, *n* = 40 per trial in all experiments.

NADPH oxidases enzymes generate superoxide anions as a product of NADPH oxidation, which are subsequently converted into H_2_O_2_ by superoxide dismutases (Bolwell, 1999). As coronatine inhibits NADPH-dependent ROS production, we considered the possibility that the toxin inhibits superoxide dismutase activity. To test it, we performed an epinephrine autoxidation assay, which generates superoxide anion that can be scavenged by epinephrine itself, leading to the formation of adrenochrome (Misra and Fridovich, 1972). We observed that coronatine failed to prevent the autoxidation of epinephrine, thus indicating that it does not act by scavenging superoxide anions (**Supplementary Figure S2**). We also found that coronatine cannot prevent stomatal closure induced by exogenously applied H_2_O_2_ (**Figure 3A**), a result consistent with the predicted inhibitory effect of coronatine on NADPH oxidases, which also implies that this compound is unlikely to act as a H_2_O_2_ scavenger. Next, we investigated the effect of coronatine on promotion of closure by darkness, which requires NADPH oxidases (Desikan et al., 2004). We observed that stomatal closure by darkness is impaired by treatment with the NADPH oxidase inhibitor DPI or in *atrbohd* and *atrbohd/f* mutants, but not by the peroxidase inhibitor SHAM (**Figures 3B,C**). ABA can partially close stomata in the presence of the NADPH oxidase inhibitor DPI or in the double NADPH oxidase mutant *atrbohd/f*, something which is believed to be due to redundancy of the complex ABA signaling network (Kwak et al., 2003). Consistently with this hypothesis, ABA closed stomata in this double mutant to a similar extent either in the absence or in the presence of coronatine (**Figure 3D**), thus showing that the presence of these NADPH oxidases is required for the effect of coronatine on stomata. However, the single *atrbohd* mutant, which displays a normal response to ABA but is unable to close stomata in response to flg22 (Kwak et al., 2003; Macho et al., 2012), was also unresponsive to coronatine for promotion of closure by ABA (**Figure 3D**). This result was unexpected, as we hypothesized that in *atrbohd* mutants coronatine should still inhibit AtRBOHF and thus partially reduce closure induced by the hormone in *atrbohd*. By contrast, mutations affecting kinases OST1 and BIK1, which respectively, phosphorylate and activate AtRBOHF (Sirichandra et al., 2009) and AtRBOHD (Kadota et al., 2014), do not affect sensitivity to coronatine for stomatal closure induced by flg22 in the case of *ost1-2*, and by ABA in the case of *bik1* (**Figures 3E,F**).

### Coronatine Reveals a Possible Regulation of ROS on ABA Signaling Components in Guard Cells

**FIGURE 4:**
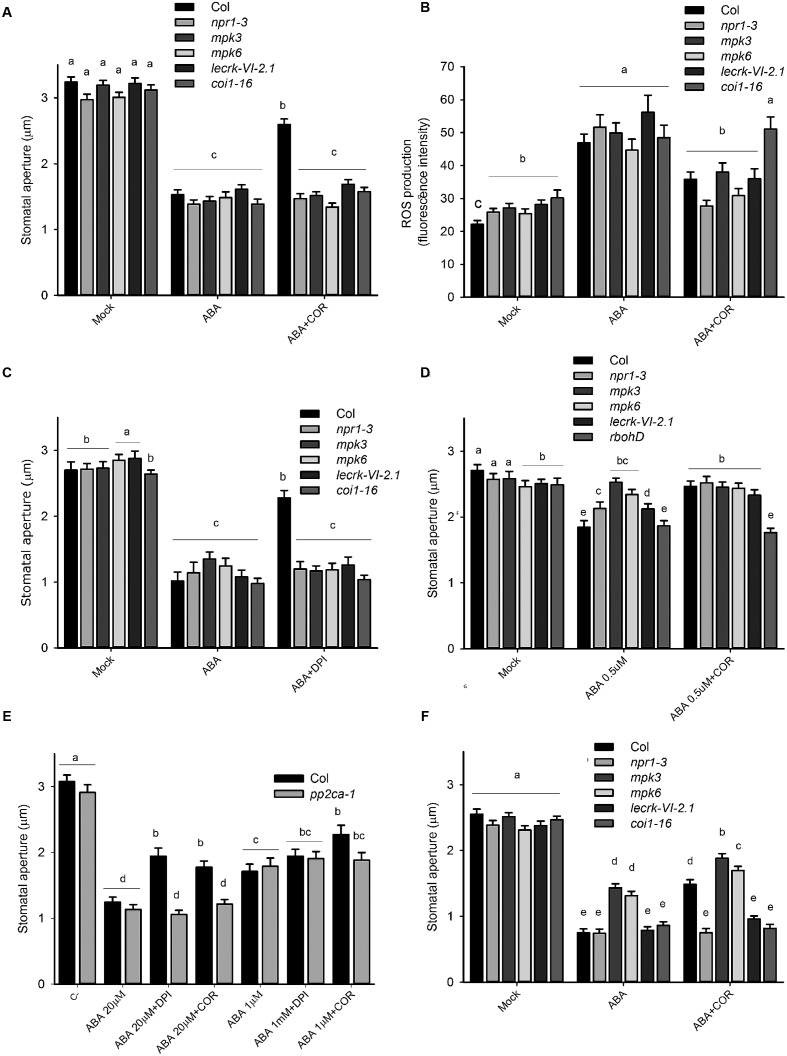
**Several mutants display reduced stomatal sensitivity to coronatine and DPI.** **(A)** Coronatine (COR, 1.56 μM) failed to inhibit stomatal closure by ABA (20 μM) in *mpk3*, *mpk6*, *npr1-3* and *lecrkVI-2.1*, and *coi1-16* mutants. **(B)** Coronatine (COR, 1.56 μM) inhibited ABA (20 μM)-induced ROS production in guard cells of *mpk3*, *mpk6*, *npr1-3*, and *lecrkVI-2-1* mutants. **(C)** Treatment with DPI (20 μM) failed to interfere with stomatal closure induced by ABA (20 μM) in *npr1-3*, *mpk3*, *mpk6*, and *lecrkVI-2-1* mutants but not in wild type Col. **(D)** *mpk3, mpk6*, *npr1-3*, and *lecrkVI.2-1* showed different degrees of insensitivity to ABA (0.5 μM). At this lower ABA concentration, coronatine (COR, 1.56 μM) prevented stomatal closure in *npr1-3* and *lecrkVI.2-1* mutants. **(E)** *pp2ca-1* mutant is insensitive to coronatine (COR, 1.56 μM) and DPI (20 μM) in stomatal closure induced by 20 μM ABA. **(F)** Coronatine (COR, 1.56 μM) partially relieved inhibition of light-induced stomatal opening by ABA (20 μM) in *mpk3*, *mpk6*, but not in *npr1-3* and *lecrkVI-2-1* mutants. DPI was added to the incubation buffer 10 min prior to ABA (20 μM). In **(A,B)** and **(D–F)** coronatine was added 10 min before ABA. In all cases stomatal apertures were measured 1.5 h after treatments. Different letters indicate significant differences at *p* < 0.05 in **(A–F**; two-way ANOVA). Error bars represent SE from two independent trials, *n* = 40 per trial **(A–F)**.

Because of the insensitivity to coronatine of *atrbohd*, we hypothesized that abnormal ROS synthesis in this mutant might lead to alterations in the ABA signaling network. To investigate this possibility, we tested the ability of coronatine to inhibit ABA-induced stomatal closure in other mutants which, like *atrbohd*, are also affected in sensitivity to flg22 but not to ABA. For this purpose, we used *mpk6*, *mpk3* (Montillet et al., 2013), *npr1-3* (Zeng and He, 2010), and *lecrkVI.2-1* (Singh et al., 2012). We observed that in all these mutants, like in *atrbohd*, this toxin was unable to inhibit closure induced by ABA (**Figure 4A**). As coronatine inhibits ROS production triggered by ABA in wild type plants, we predicted that in coronatine-insensitive mutants ABA-induced ROS synthesis in stomata would not be affected. However, we observed that coronatine did inhibit ABA-induced ROS production in *mpk3, mpk6*, *npr1-3*, and *lecrkVI.2-1* mutants in a similar way as in wild type plants (**Figure 4B**), indicating that they are affected downstream or independently from NADPH oxidase-dependent ROS production. Consistent with the role of COI1 as a receptor of coronatine, no inhibitory effect of this toxin on stomatal closure or in guard cell ROS production was observed in *coi1-16* mutant (**Figures 4A,B**). These results suggest that *mpk3, mpk6*, *npr1-3*, and *lecrkVI.2-1* mutants possess the ability to close stomata in response to ABA to the same extent as wild type plants without a requirement of ROS synthesis. In order to further test this possibility, we studied the effect of the NADPH oxidase inhibitor DPI on ABA-induced closure in these mutants and found that, similarly to coronatine, this compound inhibits closure triggered by the hormone in wild type but not in *mpk3, mpk6*, *npr1-3*, or *lecrkVI.2-1* mutants (**Figure 4C**). Consistent with an impairment of ROS signaling in these mutants, they all showed reduced sensitivity to exogenously applied H_2_O_2_ (**Supplementary Figure S3**). Interestingly, *coi1-16* mutant also closed stomata in the presence of DPI, suggesting that it may also be affected in ROS signaling, which may be linked to the fact that COI1 is required for NADPH oxidase regulation by coronatine. Nevertheless, unlike *mpk3, mpk6*, *npr1-3*, and *lecrkVI.2-1*, *coi1-16* sensitivity to exogenous H_2_O_2_ is not affected (**Supplementary Figure S3**). When 0.5 μM instead of 20 μM ABA was used, *mpk3, mpk6*, *npr1-3*, and *lecrkVI.2-1* showed different degrees of insensitivity to the hormone, which indicates abnormal ABA signaling in these mutants (**Figure 4D**), thus suggesting that proteins affected by such mutations play some role in the transduction of ROS generated by ABA in guard cells. Interestingly, in the two mutants which displayed some degree of sensitivity to 0.5 μM ABA, *npr1-3*, and *lecrk-VI.2-1*, coronatine was capable of reducing closure triggered by this low concentration of the hormone (**Figure 4D**). This result suggests that at least in *npr1-3* and *lecrk-VI.2-1* at low ABA concentrations the putative signaling components upregulated by reduced ROS signaling play a less dominant role, so that ROS signaling is more important and therefore coronatine significantly affects closure triggered by low concentrations of ABA. Consistently with this possibility, as noted above, *mpk3*, *mpk6*, *npr1-3*, and *lecrk-VI.2-1* display only partially impaired sensitivity to exogenous H_2_O_2_ in stomatal closure. Unlike the rest of the tested mutants, *rbohd* behaved in a similar way at 20 and 0.5 μM ABA, with similar sensitivity to ABA and complete insensitivity to coronatine (**Figure 4D**). This could be related to the fact that AtRBOHD is involved in ROS synthesis but not in signaling, as likely is the case of the other four mutants. Since it has been shown that both overexpression of the RCAR3 ABA receptor and mutation of the gene encoding its interacting phosphatase PP2CA also cause loss of sensitivity to coronatine for ABA-induced stomatal closure (but not reduction in sensitivity to flg22; Lim et al., 2014), we hypothesized that, according to our model of action of coronatine, these transgenic lines and mutants would also be affected in sensitivity to the NADPH oxidase inhibitor DPI. We observed that this is the case for *pp2ca-1* mutant, indicating that the reason of stomatal insensitivity to coronatine of this mutant is likely a defect in ROS signaling of synthesis (**Figure 4E**). When 1 instead of 20 μM ABA was used, no effect of DPI was observed either in *pp2ca-1* or in wild type plants. Altogether, these results suggest that ROS may negatively regulate ABA signaling components that can compensate for abnormal ROS signaling in *mpk3, mpk6*, *npr1-3* and *lecrkVI.2-1*, and possibly also in NADPH oxidase mutants *atrbohD* and *atrbohF*.

Coronatine interferes also with inhibition of light-induced stomatal opening by flg22 by relieving its inhibitory effect on K^+^_in_ currents (Zhang et al., 2008). Since there is strong evidence indicating that the ABA signaling network is different for inhibition of opening and for promotion of closure (Yin et al., 2013), we tested if *mpk3*, *mpk6*, *npr1-3*, and *lecrkVI.2-1* mutants are similarly affected in sensitivity to coronatine in assays of inhibition of light-induced stomatal opening by ABA. Just as observed in promotion of closure experiments, coronatine failed to prevent inhibition of stomatal opening by ABA in *npr1-3* and *lecrkVI.2-1* (**Figure 4F**). As previously reported, *mpk3* mutants (Gudesblat et al., 2007), and also *mpk6*, displayed partial insensitivity to ABA for inhibition of light-induced stomatal opening. Both mutants were only partially insensitive to coronatine. As expected, *coi1-16* mutants were also insensitive to coronatine, and responded like wild type to ABA. These results indicate that the signaling components affected by coronatine which are required for ROS synthesis are active in both promotion of closure and inhibition of opening by ABA.

## Discussion

Our results show a mechanism through which coronatine, a phytotoxin produced by the pathogenic bacterium *Pst* DC3000, hijacks stomatal immunity, through inhibition of guard cell NADPH oxidase-dependent ROS production. Coronatine inhibits closure by flg22, ABA and darkness, which trigger ROS production through NADPH oxidases. However, it does not affect peroxidase-dependent ROS production or stomatal closure triggered by SA, which promotes stomatal closure through apoplastic peroxidases. In addition, coronatine does not inhibit the oxidative burst triggered by flg22 in leaf disks, suggesting that it affects a guard-cell specific regulatory mechanism of AtRBOHD and AtRBOHF, and that it does not directly inhibit their enzymatic activity. NADPH oxidases generate as a reaction product the short lived superoxide anion, which is enzymatically dismutated into the more stable H_2_O_2_ derivative that is required for a viable long-range cell-to-cell signal, for passing membranes or for accumulation. This step occurs very quickly and is catalyzed by superoxide dismutase (Sagi and Fluhr, 2006). Thus an inhibitory effect of coronatine on superoxide dismutases cannot be completely ruled out. By contrast, coronatine does not appear to act as a ROS scavenger, as it cannot prevent stomatal closure by exogenously applied H_2_O_2_ or *in vitro* epinephrine autoxidation, which generates superoxide anion.

Reactive oxygen species act as important signaling hubs in guard cell signaling (Baxter et al., 2014; Kollist et al., 2014), and therefore their concentration is tightly regulated. NADPH oxidases AtRBOHD and AtRBOHF have been shown to be regulated in multiple ways, including direct binding of Ca^2+^ (Ogasawara et al., 2008) and phosphatidic acid (Zhang and Du, 2009), and phosphorylation by OST1/SnRK2.6 (Sirichandra et al., 2009), CBL-interacting protein kinase CIPK26 (Drerup et al., 2013), calcium dependent protein-kinase 5 (Dubiella et al., 2013) and the kinase BIK1, which is part of the receptor complex for several MAMPs (Kadota et al., 2014; Li et al., 2014). Coronatine could thus interfere with some of these regulatory mechanisms to regulate NADPH oxidase activity in guard cells. Mutations in BIK1 and OST1 do not affect sensitivity to coronatine, which seems to rule out the possibility that the effect of the toxin is mediated by these kinases. Consistently with our findings, it has been reported that coronatine reversed the inhibitory effect of flg22 on guard cell K^+^_in_ currents (Zhang et al., 2008), which in turn are negatively regulated by H_2_O_2_ (Kohler et al., 2003).

Coronatine facilitates *Pst* DC3000 invasion in several ways, including inhibition of stomatal immunity, promotion of bacterial multiplication and persistence inside the plant. It also causes induction of disease symptoms, enhancement of disease susceptibility in uninfected parts of the plant, inhibition of cell wall defenses and a delay in hypersensitive response cell death (Lee et al., 2013; Xin and He, 2013; Gimenez-Ibanez et al., 2016). However, none of these effects of coronatine have been linked to inhibition of NADPH oxidase-dependent ROS synthesis. Instead, coronatine treated plants displayed increased ROS production after 24 h both in tomato (Ishiga et al., 2009) and in *A. thaliana* (Camanes et al., 2012) whole leaves. Recently it has been shown that coronatine strongly induces the expression of N-ACETYLTRANSFERASE ACTIVITY1 (NATA1), which leads to decreased defense-related H_2_O_2_ accumulation through interference with polyamine metabolism (Lou et al., 2016). However, this effect occurs a few hours after coronatine treatment, while the effect of coronatine on stomata is very fast. Interestingly, some pathogen effectors have been shown to promote virulence by interfering with ROS induction. For example, the *Ustilago maydis* fungal effector Pep1, contributes to the penetration of the host epidermis by inhibiting apoplastic plant peroxidases (Hemetsberger et al., 2012), while the *Phytophthora sojae* effector CRN 115 decrease H_2_O_2_ accumulation during infection through interaction with plant catalases (Zhang et al., 2015).

**FIGURE 5:**
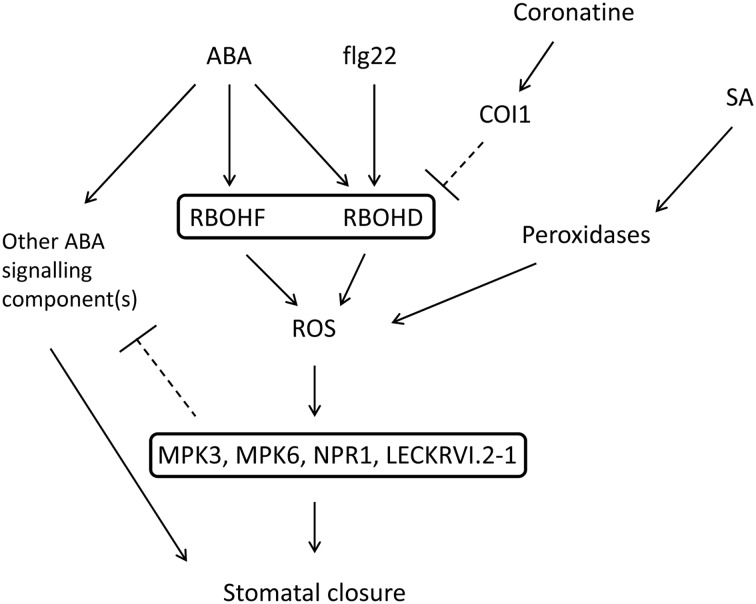
**Proposed model of ROS inhibition by coronatine in the context of ABA signaling.** Coronatine inhibits stomatal closure induced by ABA and flg22 through inhibition of ROS synthesis by NADPH oxidases AtRBOHD and AtRBOHF. This toxin does not affect promotion of stomatal closure by SA, which induces ROS synthesis mediated by peroxidases. In *atrbohd*, *mpk3*, *mpk6*, *npr1-3*, and *lecrkVI-2.1* mutants the sensitivity to ABA is not affected by coronatine or DPI. This might be due to negative regulation by ROS of other ABA signaling components, which would be upregulated in these mutants. Thus in these mutants ABA is able to close stomata in the absence of ROS signaling.

Testing of coronatine on stomata of different mutants affected in stomatal responses to flg22 but not to ABA in order to better understand the mechanism of action of the toxin allowed us to find unexpected responses to this hormone in them. The double NADPH oxidase mutant *atrbohd/f*, incapable of synthesizing ROS in stomata in response to ABA (Kwak et al., 2003), is insensitive to coronatine, consistently with the proposed inhibitory effect of this toxin on ROS production by NADPH oxidases. However, the single mutant *atrbohd*, affected in stomatal response to flg22 but not to ABA, turned out to be unresponsive to coronatine for ABA-induced promotion of closure. This finding surprised us, since we reasoned that inhibition of intact AtRBOHF by coronatine in *atrbohd* should cause reduced stomatal response of this mutant to ABA. Previous reports have described unexpected phenotypes in *atrbohd* mutant, which display constitutive or inducible over-activation of immunity (reviewed in Kadota et al., 2015). In order to test whether lack of response to coronatine is specific of *atrbohd*, or if by contrast it is also found in other mutants unresponsive to MAMPs in guard cells, we analyzed *mpk3*, *mpk6*, *npr1-3*, and *lecrkVI.2-1* mutants, which like *atrbohd*, are affected in stomatal sensitivity to ABA but not to flg22. Similarly to *atrbohd*, they also displayed insensitivity not only to coronatine but also to the NADPH oxidase inhibitor DPI for promotion of closure by ABA. This hormone was capable of inducing ROS synthesis in guard cells of all four mutants and, somewhat surprisingly, coronatine was still capable of inhibiting it, even when the toxin could not inhibit the stomatal response to the hormone in the mutants. These findings made us suspect that, unlike wild type plants, *mpk3*, *mpk6*, *npr1-3*, and *lecrkVI.2-1* mutants do not require ROS for ABA-induced promotion of closure. This hypothesis was strengthened by the reduced sensitivity to stomatal closure triggered by exogenous H_2_O_2_ in these mutants, a result suggesting that MPK3, MPK6, NPR1-3, AND LECRKVI.2-1 are involved in signaling downstream of ROS. Thus, we propose that guard cells ABA signaling components independent of ROS might compensate for defects in ROS synthesis or signaling such as those existing in the analyzed mutants. The synthesis of such ROS-independent signaling components would be negatively regulated by ROS in the long term, and thus upregulated in *mpk3*, *mpk6*, *npr1-3*, and *lecrkVI.2-1*, and likely also in *atrbohd* (**Figure 5**). Interestingly, it was previously reported that a guard cell-specific *MPK3* antisense mutant, displaying a stomatal phenotype similar to the *mpk3* mutant used in this work, is insensitive to inhibition of ABA-induced stomatal closure by a virulence factor secreted by *Xanthomonas campestris* pv. *campestris* with a similar effect to coronatine on stomata (Gudesblat et al., 2009). Reduced sensitivity to coronatine was also observed for inhibition of opening by ABA, even when signaling in this process differs from that of promotion of closure by the same hormone (Yin et al., 2013). Interestingly, *mpk3* and *mpk6* mutants were partially insensitive to ABA, a phenotype previously reported in guard cell-specific *MPK3*⋅antisense lines (Gudesblat et al., 2007). In both mutants coronatine can further reduce ABA sensitivity, suggesting that the diminished sensitivity of *mpk3* and *mpk6* to ABA is in part independent of the NADPH oxidases targeted by coronatine. Further support for our proposal that coronatine affects ROS synthesis comes from the insensitivity of *pp2ca-1* mutant to the NADPH oxidase inhibitor DPI. Unlike *mpk3*, *mpk6*, *npr1-3* and *lecrkVI.2-1*, *pp2ca-1* is exclusively affected in ABA signaling, and has been shown to display a wild type stomatal respose to flg22 (Lim et al., 2014). Thus, while the insensitivity to coronatine of *pp2ca-1* has probably a different origin than that of *mpk3*, *mpk6*, *npr1-3*, and *lecrkVI.2-1*, still all of them share DPI insensitivity, strongly linking the effect of coronatine to NADPH-dependent ROS signaling. *pp2ca-1* mutants are hypersensitive to ABA in promotion of stomatal closure (Kuhn et al., 2006), which suggests that increased ABA signaling in this mutant somehow inhibits either stomatal ROS production in response to the hormone or sensitivity to them (Kuhn et al., 2006).

How MPK3, MPK6, NPR1, and LECKVI.2 might precisely act in ABA and MAMP signaling is not completely clear, but this possibility is strengthened by our observation that mutants affected in all four of them displayed reduced sensitivity to ABA when 0.5 μM instead of 20 μM was used. MPK3 and MPK6 are generally believed to act downstream of ROS signaling, however, there is also evidence that these enzymes might act upstream or independently from ROS (reviewed in Jalmi and Sinha, 2015). NPR1 is a transcription factor essential for SA signaling which has previously been proposed to act upstream of ABA, given that ABA biosynthesis is required for SA-mediated stomatal closure (Zeng and He, 2010). It has also been proposed that coronatine inhibits stomatal closure through inhibition of SA synthesis (Zheng et al., 2012), but our results do not support this hypothesis, as coronatine still inhibits ROS induction by ABA even when SA signaling is disrupted in *npr1-3* mutants in guard cells. Furthermore, these mutants display reduced sensitivity to low concentrations of the hormone, which would not be expected if NPR1 acts upstream of ABA synthesis. LECRKVI.2 is an L-type lectin receptor kinase required for flg22-induced MPK3 and MPK6 activation and for pathogen resistance (Singh et al., 2012). How LECRKVI.2 might precisely act in signaling downstream of ROS also needs to be clarified. The loss of function *lecrk-V.5* mutants, affected in a related receptor, are less sensitive to coronatine (Desclos-Theveniau et al., 2012). This could be due to their enhanced ROS synthesis, which might compensate for the inhibition of ROS production caused by this toxin in guard cells.

The importance of JA signaling network in antagonizing stomatal closure by MAMPs is highlighted by the fact that *Pseudomonas syringae* pv. *tabac*i effector HopX1 inhibits stomatal closure through activation of JA signaling by promoting the degradation of JASMONATE ZIM-domain transcriptional repressors proteins (JAZ), and can complement a *Pst* coronatine deficient strain (Gimenez-Ibanez et al., 2014). Recently it has also been shown that the *Pst* effector protein AvrB can rescue the capacity of opening stomata of a *Pst* coronatine deficient strain in a COI1-dependent manner (Zhou et al., 2015). However, while it is clear that coronatine inhibit stomatal closure triggered by flg22 and ABA, evidence regarding the effect of jasmonates on stomata is conflicting. While some reports have shown that MeJA promotes stomatal closure (Gehring et al., 1997; Suhita et al., 2004), other groups have found either no effect of this compound on stomatal closure (Zhao et al., 2008), or an inhibitory effect on flg22-induced stomatal closure in a JA signaling-independent manner (Montillet et al., 2013). Although different jasmonate forms exist in plants, only the conjugate JA-Ile and its structural analog coronatine produced by *Pst* are known to be biologically active. Both of them, but not racemic (±) JA, can promote opening of dark-closed *Ipomoea tricolor* stomata (Okada et al., 2009). It might thus be possible that under different environmental conditions or in different tissues MeJA or other jasmonates are conjugated differently inside the cell, leading to compounds with different activities.

Our work shows that coronatine exerts its inhibitory effect on stomata by affecting ROS synthesis mediated by NADPH oxidases AtRBOHD and AtRBOHF and thus reinforces the important role of NADPH oxidases in guard cell signaling. Understanding how coronatine precisely causes this effect might help to develop strategies to prevent the entry of *Pst* through stomata.

## Author Contributions

GG and LT conceived the idea of this work, executed experiments, and wrote the manuscript. MB and SG designed and executed superoxide measurement experiments. AV and PT contributed with discussion and critical comments. All authors approved the final version.

## Conflict of Interest Statement

The authors declare that the research was conducted in the absence of any commercial or financial relationships that could be construed as a potential conflict of interest.
